# Hyponatremia During Induction Therapy in Distinct Pediatric Oncological Cohorts: A Retrospective Study

**DOI:** 10.3389/fonc.2021.708875

**Published:** 2021-10-29

**Authors:** Christina Salvador, Robert Salvador, Peter Willeit, Christine Kuntner, Alexandra Haid, Thomas Müller, Gabriele Kropshofer, Roman Crazzolara

**Affiliations:** ^1^ Department of Pediatrics I, Division of Hematology and Oncology, Medical University Innsbruck, Innsbruck, Austria; ^2^ HTLinn, Innsbruck, Austria; ^3^ Department of Neurology and Neurosurgery, Medical University Innsbruck, Innsbruck, Austria; ^4^ Department of Public Health and Primary Care, University of Cambridge, Cambridge, United Kingdom; ^5^ Information Technology Management, Medical University Innsbruck, Innsbruck, Austria; ^6^ Competence Center for Clinical Studies, Medical University Innsbruck, Innsbruck, Austria

**Keywords:** alkaloids, children, hyponatremia, leukemia, triglycerides, vincristine

## Abstract

**Background:**

Hyponatremia is a well-known adverse event of repeated therapy with vincristine in oncological patients. However, to date, data in pediatric patients with malignant diseases other than acute lymphoblastic leukemia (ALL) are sparse or lacking.

**Materials and Methods:**

A retrospective study of 98 pediatric patients was conducted to analyze the incidence of hyponatremia in a Caucasian cohort of newly diagnosed ALL. For comparison, we further examined five other pediatric oncological cohorts (Hodgkin’s disease, Ewing sarcoma, Wilms tumor, benign glioma of the CNS, Langerhans cell histiocytosis) that receive alkaloids in their induction regimes.

**Results:**

We found a high incidence of hyponatremia (14.7%) in our ALL cohort with a trend toward male patients of elementary school age. None of the affected patients showed neurological symptoms. By comparison, patients from other malignancy groups did not show significant hyponatremia, regardless of their comparable therapy with alkaloids. We here show a noticeable coincidence of hyponatremia and hypertriglyceridemia in ALL patients, indicating a possible role of L-asparaginase-related hypertriglyceridemia in the development of severe hyponatremia in such patients.

**Conclusion:**

We report a higher incidence of hyponatremia following vincristine therapy in Caucasian children with ALL than published before. This hyponatremia could not be demonstrated in other oncologic cohorts treated with alkaloids. L-Asparaginase-induced hypertriglyceridemia may play a role in the certainly multifactorial development of hyponatremia in childhood leukemia.

## Introduction

Hyponatremia is an electrolyte disturbance commonly seen in clinical practice. Its etiology is heterogeneous. The syndrome of inappropriate secretion of antidiuretic hormone (SIADH) is the most common underlying cause. It can occur as a paraneoplastic phenomenon, as a result of central nervous system (CNS) lesions (intracranial tumor masses or metastatic lesions, meningitis, or cranial irradiation) or of pulmonary disease (pneumonia, metastasis), or as an adverse effect of anticancer therapy (1–4). Among vinca alkaloids, vincristine and, to a lesser extent, vinblastine have been frequently reported to trigger hyponatremia in oncological patients through a direct neurotoxic effect on sites in the hypothalamus, the neurohypophyseal tract, or the posterior pituitary gland (5–20). This neurotoxic effect of vincristine has also been demonstrated in animal models (21, 22). Vinca alkaloids are indicated for the treatment of a variety of pediatric oncological diseases, among them acute lymphoblastic leukemia (ALL), Hodgkin’s or non-Hodgkin’s lymphoma, sarcoma, and some other tumor entities. Severe hyponatremia of rapid onset (<48 h) is a medical emergency that can lead to serious neurological complications, which may be therapy-delaying or even life-threatening. Only sparse data address hyponatremia during intensive therapy of pediatric oncological patients. In this retrospective study, we focus on the occurrence of severe hyponatremia during induction chemotherapy with vinca alkaloids in a pediatric Caucasian cohort of ALL patients. For comparison, we analyze five additional similarly treated cohorts of pediatric patients with oncological diagnoses other than ALL.

## Materials and Methods

### Patients Included in the ALL-BFM 2009 Protocol

**Figure 1:**
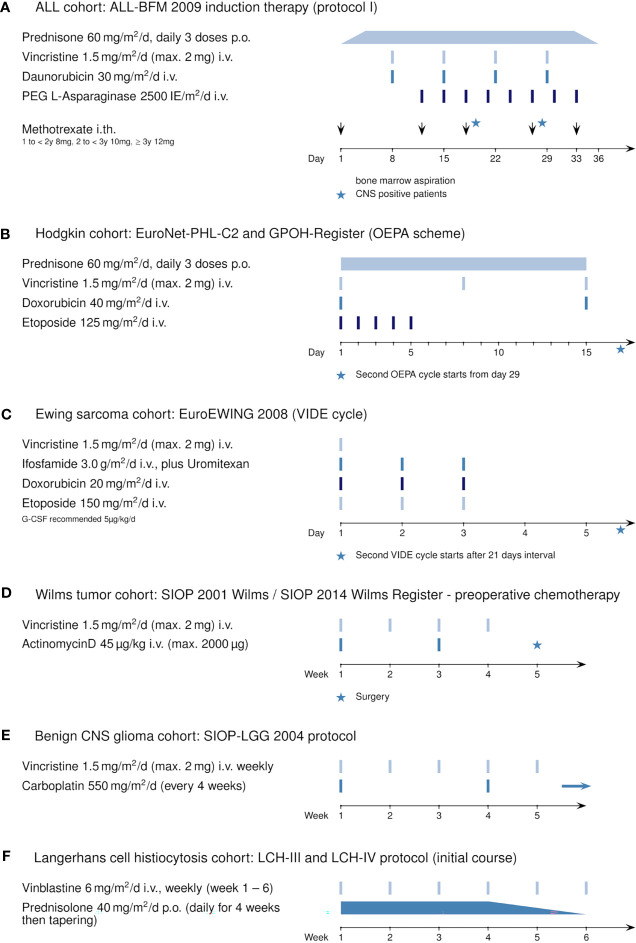
Induction therapy protocols using vincristine or vinblastine: **(A)** ALL cohort: ALL-BFM 2009 (Protocol I, induction), **(B)** Hodgkin’s cohort: EuroNet-PHL-C2 protocol and GPOH Register (OEPA scheme), **(C)** Ewing sarcoma cohort: EuroEWING 2008 (VIDE cycle), **(D)** Wilms tumor cohort: SIOP 2001 Wilms and SIOP 2014 Wilms Register (preoperative chemotherapy), **(E)** benign CNS glioma cohort: SIOP-LGG 2004, and **(F)** Langerhans cell histiocytosis cohort: LCH-III and LCH-IV protocol.

The retrospectively evaluated principal cohort included 98 children and adolescents (age >1 year to <18 years) with ALL newly diagnosed at our institution (Department of Pediatrics I, Medical University of Innsbruck) between December 2010 and January 2019. Three patients were excluded from analysis because transfer to another hospital resulted in absence of serum sodium values for at least 10 consecutive days. Of the remaining 95 patients, 56 were male (58.9%) and 39 were female (41.1%). The mean age at diagnosis was 6.3 years (range 1.1 to 17.9 years, see also **Table 1**). All patients received chemotherapy according to the ALL-BFM 2009 protocol (**Figure 1A**, https://www.clinicaltrialsregister.eu/ctr-search/trial/2007-004270-43/DE). Relevant for this study is the weekly intravenous administration of vincristine during induction therapy (four doses, starting on day 8).

**Table 1:** General statistical information on patients included in the analyzed studies (age and gender distributions).

Diagnosis	Inclusion period	No. of patients	Male	Female	Age at diagnosis
**ALL cohort**	12/2010–01/2019	95 (3/98 excluded)	56 (58.9%)	39 (41.1%)	6.3 years (1.1–17.9)
**Hodgkin’s disease**	From 08/2013 (still open)	28 (2/30 excluded)	12 (42.9%)	16 (57.1%)	13.5 years (5.0–18.0)
**Ewing sarcoma**	05/2010–06/2019	23	11 (47.8%)	12 (52.2%)	11.9 years (3.0–18.1)
**Wilms tumor**	From 10/2010 (still open)	20	6 (30.0%)	14 (70.0%)	3.5 years (0.5–14.8)
**Benign CNS glioma**	From 04/2004 (still open)	16 (1/17 excluded)	7 (43.8%)	9 (56.2%)	3.6 years (0.4–11.5)
**Langerhans cell histiocytosis**	From 04/2001 (still open)	9	5 (55.6%)	4 (44.4%)	5.1 years (0.2–11.6)

All high-risk patients received antifungal prophylaxis with liposomal amphotericin-B instead of formerly used azoles. Patients not meeting criteria for high-risk status received no routine fungal prophylaxis at all.

### Patients Included in Therapy Protocols Other Than ALL-BFM 2009

Additionally, for comparison, we retrospectively analyzed five pediatric oncological cohorts in which patients received vincristine (or vinblastine) during their first weeks of treatment: 28 patients with newly diagnosed Hodgkin’s disease included in the EuroNet-PHL-C2 and the GPOH Register therapy protocol (**Figure 1B**, OEPA scheme); 23 patients with newly diagnosed Ewing sarcoma enrolled in the EuroEWING 2008 protocol (**Figure 1C**, VIDE cycle); 20 patients with Wilms tumor during preoperative chemotherapy (**Figure 1D**, SIOP 2001 Wilms/SIOP 2014 Wilms Register); 16 patients with initial diagnosis of a benign glioma of the CNS (**Figure 1E**, SIOP-LGG 2004); and 9 patients with Langerhans cell histiocytosis (**Figure 1F**, LCH-III/-IV). For information on the general statistics, age distribution, and gender distribution of these patient groups, see **Table 1**.

During the first days of induction chemotherapy, to prevent tumor lysis syndrome, all newly diagnosed patients with great tumor burden (especially those with ALL, Hodgkin’s disease, Ewing sarcoma, or Wilms tumor) received hydration therapy consisting of a semi-isotonic solution (50% NaCl 0.9%, 50% glucose 5%, 40 ml/l sodium bicarbonate) administered intravenously at 3 l/m^2^/24 h.

### Laboratory Methods

Routine blood specimens for electrolyte determinations were drawn daily or at least every 2 to 3 days during induction chemotherapy (first 40 days). If hyponatremia was detected, blood specimens were drawn at least twice a day or even more often until serum sodium values normalized. Serum sodium was detected by indirect potentiometry in our central laboratory (Innsbruck Medical University Hospital). The standard range of serum sodium in a healthy child is 132 to 145 mmol/l (23). Severe hyponatremia (a “hyponatremic episode”) was defined as a serum sodium level ≤130 mmol/l on at least 2 of 3 consecutive days.

### Statistical Analysis

**Figure 2:**
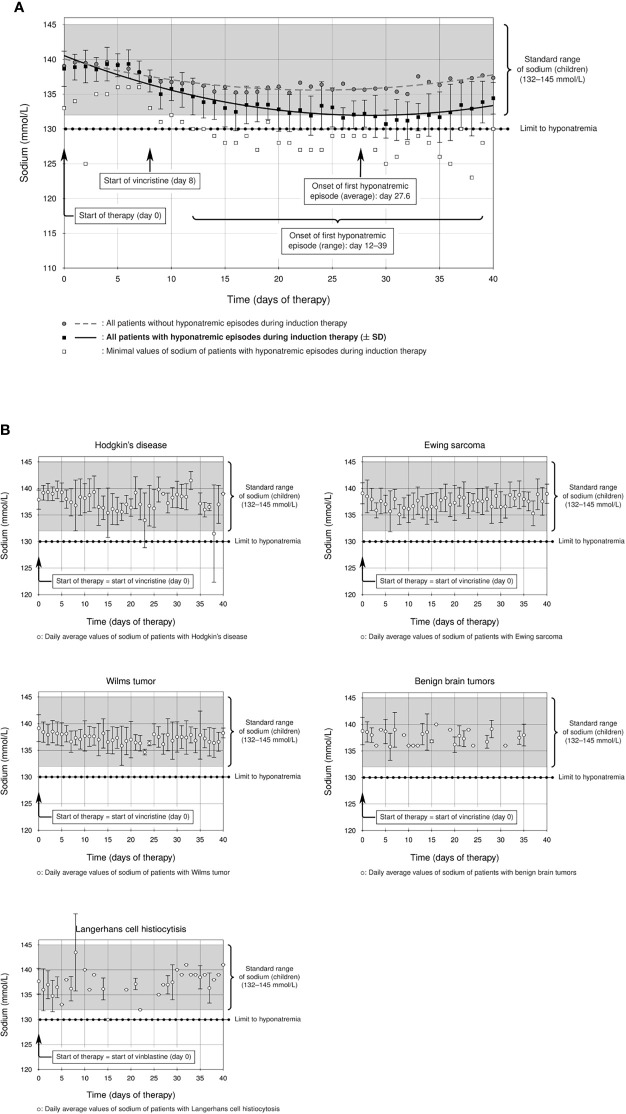
**(A)** Prevalence of hyponatremia in the studied ALL cohort: a considerable group of ALL patients showed statistically significant hyponatremia during induction therapy (solid line, ± standard deviation) compared to the rest of the ALL cohort (dashed line), which remained within the standard range of sodium in childhood (132–145 mmol/l). The start of the first hyponatremic episode was on average between days 27 and 28 (average day 27.6, curly brackets), although there was a statistically significant difference between hyponatremic and non-hyponatremic patients from day 12 (p = 0.014, F test and T test, curly brackets). **(B)** Mean sodium levels of all patients with the following diagnoses during the first 40 days of their initial therapy. Patients with Hodgkin’s lymphoma, Ewing sarcoma, Wilms tumor, benign brain tumors, and Langerhans cell histiocytosis do not show hyponatremia during therapy with alkaloids (sodium levels within standard range).

Analysis of primary study endpoints was performed with descriptive and inferential statistics. The measured sodium values of all patients on a particular day of treatment were summarized in two calculated values for that day: the minimum sodium values of all patients were averaged (= MIN) as were the mean sodium values of all patients (= MEAN). However, not every patient necessarily had a sodium value on every single day. On the other hand, some patients had two or more sodium values on some days. The measuring dots in the diagrams (**Figure 2**) thus result from the above-indicated day/MIN pairs and day/MEAN pairs (for both hyponatremic and non-hyponatremic patients). Data analysis and statistical tests were performed with a C++ program (version C++17). Statistically significant differences between the sodium levels of hyponatremic and non-hyponatremic patients were determined with statistics F tests and subsequent T tests for all days of treatment. p values and 95% confidence intervals for the differences between the mean values were calculated. Statistical associations between hyponatremia and, individually, age, gender, CNS status, high-risk status, hypertriglyceridemia, and hyperglycemia were calculated using Fisher’s exact test. p values below 0.05 were deemed statistically significant. Graphic presentation was performed with LaTeX (version TeX Live 2020).

### Ethics

The Ethics Committee of the Medical University of Innsbruck approved the retrospective evaluation (EC No. 1477/2020). All data were obtained from medical records. This study was performed in accordance with the Declaration of Helsinki.

## Results

### Statistically Significant Severe Hyponatremia in Caucasian Children With ALL

The incidence of severe hyponatremia during ALL-BFM 2009 induction therapy was 14.7% (14/95 cases, **Table 2**) in our Caucasian cohort, a value higher than those reported for pediatric ALL cohorts (24, 25). The difference between hyponatremic and non-hyponatremic patients was statistically significant from day 12 after the start of therapy (**Figure 2A**, day 12, p = 0.014, **Supplemental Table**). The average age of hyponatremic patients was 9.0 (range 2.2–17.9) years, whereas the average age of non-hyponatremic patients was lower (5.9 years, range 1.1–17.5). Divided according to age groups, three hyponatremic patients (21.4%) were aged <5 years, 7 (50%) 5–10 years, none 10–13 years, and 4 (28.6%) >13 years. Hyponatremic patients were significantly older than non-hyponatremic patients (p = 0.0369, **Table 2**). Furthermore, 10 (71.4%) of the 14 hyponatremic patients were male, while only 4 (28.6%, **Table 2**) were female. However, no statistically significant difference could be shown regarding gender (p = 0.385, **Table 2**).

**Table 2:** Prevalence of hyponatremia in the examined ALL cohort (14.7%).

Relevant factors	Hyponatremia	Normal sodium level	Fisher’s exact test
**ALL cohort**	14/95 (14.7%)	81/95 (85.3%)	
**Age group**			
< 5 years	3/14 (21.4%)	47/81 (58.0%)	p = 0.0369
5–10 years	7/14 (50.0%)	23/81 (28.4%)	
10–13 years	0/14 (0.0%)	2/81 (2.5%)	
>13 years	4/14 (28.6%)	9/81 (11.1%)	
Average age (years)	9.0 (range: 2.2–17.9)	5.9 (range: 1.1–17.5)	
**Gender**			
Male	10/14 (71.4%)	46/81 (56.8%)	p = 0.385
Female	4/14 (28.6%)	35/81 (43.2%)	
**CNS status**			
Negative	12/14 (85.7%)	76/81 (93.8%)	p = 0.274
Positive	2/14 (14.3%)	5/81 (6.2%)	
**Risk group**			
Non-high risk	9/14 (64.3%)	65/81 (80.3%)	p = 0.292
High risk	5/14 (35.7%)	16/81 (19.7%)	
**Triglycerides**			
>1000 mg/dl	6/14 (42.9%)	4/81 (4.9%)	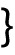 p = 0.000177
>500 mg/dl	1/14 (7.1%)	14/81 (17.3%)	
Normal	3/14 (21.4%)	53/81 (65.4%)	
No data available	4/14 (28.6%)	10/81 (12.4%)	
**Serum-protein**			
**• During hyponatremia**			
> 8 g/dl	0/14 (0.0%)	–	
Normal	2/14 (14.3%)	–	
<5 g/dl	12/14 (85.7%)	–	
**• After asparaginase**			
>8 g/dl	0/14 (0.0%)	0/81 (0.0%)	
Normal	0/14 (0.0%)	24/81 (29.6%)	
<5 g/dl	14/14 (100.0%)	57/81 (70.4%)	
**Serum-glucose**			
**• During hyponatremia**			
>150 mg/dl	2/14 (14.3%)	–	
<150 mg/dl	12/14 (85.7%)	–	
**• During induction therapy**			
>150 mg/dl	4/14 (28.6%)	28/81 (34.6%)	p = 0.767
<150 mg/dl	10/14 (71.4%)	53/81 (65.4%)	

Various factors that could possibly be related to hyponatremia were examined for their statistical significance, namely, age, gender, CNS status, risk group, and relevant laboratory parameters (serum-triglycerides, serum-protein, serum-glucose). We were able to show a trend to male patients of primary school age (age p = 0.0369, gender p = 0.385). Hyponatremia is not associated with positive CNS status (p = 0.274) or high-risk disease (p = 0.292). Interestingly, a statistically significant coincidence of elevated triglycerides (>1000 mg/dl) and hyponatremia during ALL induction therapy (p = 0.00017) was observed. At least 42.9% of hyponatremic patients showed massively elevated triglycerides during hyponatremia, whereas only 4.9% of non-hyponatremic ALL patients showed hypertriglyceridemia. On the other hand, serum protein and serum glucose were not responsible for a possible pseudohyponatremia: 57/81 (70.4%) non-hyponatremic patients showed hypoproteinemia during induction therapy and 12/14 (85.7%) hyponatremic patients showed hypoproteinemia at the time of hyponatremia. No patient was observed with hyperproteinemia. Regarding serum glucose, we found 28/81 (34.6%) non-hyponatremic patients with hyperglycemia during induction therapy and only 2/14 (14.3%) hyponatremic patients with glucose levels >150 mg/dl at the time of hyponatremia. Therefore, hyperglycemia is not associated with hyponatremia (p = 0.767).

The lowest sodium level measured was 123 mmol/l in one patient. Of our hyponatremic patients, 71.4% (10/14) had only one hyponatremia episode throughout induction therapy. One patient had two episodes, another had three, and two each had four episodes during induction. The first hyponatremic episode started on average between days 27 and 28 (average day 27.6) but occurred earlier or later in some patients (ranging from day 12 to day 39 after start of therapy). A significant difference from non-hyponatremic patients thus was demonstrated as early as day 12 (**Figure 2A**, p = 0.014, **Supplemental Table**). The median duration of hyponatremia episodes was 3 days (median, range 2–6 days).

### No Statistically Significant Occurrence of Hyponatremia Episodes During Induction Therapy in Patients Other Than the ALL Group

For comparison, we analyzed other cohorts of pediatric patients with newly diagnosed hemato-oncological diseases, all of whom received vinca alkaloids (vincristine or vinblastine) in their first weeks of induction therapy (**Figures 1B–F**). Dosage regimens deviated somewhat from the usual schedule of weekly vincristine doses: Patients with Ewing sarcoma received only one dose of vincristine at the start of therapy, and patients with Langerhans cell histiocytosis were treated with weekly vinblastine instead of vincristine. We found that patients with Ewing sarcoma, Wilms tumor, or Langerhans cell histiocytosis did not show any sodium disturbances during their induction therapy (**Figure 2B**, mean sodium levels remained within the standard range; see also **Table 3**). In the patient cohorts with Hodgkin’s lymphoma and benign tumors of the CNS, one patient each was detected with one episode of hyponatremia (**Table 3**). In the Hodgkin’s group, the single hyponatremic patient was a 5-year-old girl, and in the cohort with benign CNS glioma the single patient was a 1.7-year-old boy.

**Table 3:** Difference between ALL patients and patients with other diagnoses studied in this study (Hodgkin’s disease, Ewing sarcoma, Wilms tumor, benign CNS glioma, and Langerhans cell histiocytosis) regarding occurrence of hyponatremia during induction chemotherapy.

	Hyponatremia	Normal sodium level
**ALL cohort**	14/95 (14.7%)	81/95 (85.3%)
**Hodgkin’s disease**	1/28 (3.6%)	27/28 (96.4%)
**Ewing sarcoma**	0/23 (0.0%)	23/23 (100.0%)
**Wilms tumor**	0/20 (0.0%)	20/20 (100.0%)
**Benign CNS glioma**	1/16 (6.3%)	15/16 (93.7%)
**Langerhans cell histiocytosis**	0/9 (0.0%)	9/9 (100.0%)

### Association Between Severe Hyponatremia and Other Relevant Factors in ALL Patients

We were not able to prove a statistically significant association between hyponatremia and CNS ALL involvement (p = 0.274) or between hyponatremia and high-risk status (p = 0.292, **Table 2**). Interestingly, a relatively high percentage of hyponatremic patients (42.9%, 6/14) showed significantly increased serum triglyceride values (>1,000 mg/dl) simultaneously with the hyponatremic episode, whereas only 4.9% (4/81) of non-hyponatremic ALL patients had triglyceride values >1,000 mg/dl during induction therapy. This established a statistically highly significant association between hyponatremia and hypertriglyceridemia in our ALL cohort (p = 0.00017, **Table 2**). We infer that some instances of hyponatremia thus may have been diagnosed because of an artifact of measurement, representing “pseudohyponatremia.” Other possible reasons for the development of pseudohyponatremia, namely, hyperglycemia or hyperproteinemia, can be excluded in our ALL cohort, because nearly all hyponatremic ALL patients had normal serum glucose levels at the time of hyponatremia (12/14 patients had normal glucose levels, 2/14 had only slightly increased serum glucose, **Table 2**, p = 0.767) and a high percentage showed reduced serum protein values, with serum protein levels <5 g/dl in 12/14 (85.7%) hyponatremic patients (**Table 2**) but no hyperproteinemia during hyponatremia.

### Clinical Presentation of Hyponatremic Patients

Clinically, our hyponatremic patients had no relevant serious, especially neurological, complications. Relatively nonspecific side effects such as lethargy or weakness during intensive induction chemotherapy are difficult to attribute exclusively to hyponatremia and can have various other causes, both therapy- and illness-related. Of the patients with hyponatremia, 57.1% (8/14) did not require any electrolyte or infusion therapy because they lacked symptoms or signs; their “illness” was self-limited. Three hyponatremic patients (21.4%) received one or at most two isotonic infusions in a maintenance dose. Following a very rapid decline in serum sodium levels, the final three patients received sodium chloride 1 M (molar) as a substitute for sodium at a dose of 1 mmol/kg as an isotonic infusion for 1 day each in the absence of neurological symptoms. No patient underwent fluid restriction in our hyponatremic cohort.

## Discussion

Electrolyte disturbances are common during cytotoxic therapy of malignant diseases. Some chemotherapeutic agents trigger hyponatremia, with SIADH invoked. Implicated are vinca alkaloids (vincristine, vinblastine), cyclophosphamide, ifosfamide, and platinum-based drugs as well as monoclonal antibodies and small-molecule inhibitors (6, 26, 27). Only a few reports exist of marked hyponatremia associated with anti-neoplastic therapy in children. A retrospective study from Poland identified severe hyponatremia in 11.9% of a cohort of children with acute lymphoblastic leukemia treated using various regimens, which included vinca alkaloids (24). A prospective study from India identified marked hyponatremia/SIADH in 10.8% of a cohort of children with acute lymphoblastic leukemia treated with vincristine (25). In our retrospective study, we analyzed a cohort of 95 children with ALL who were uniformly treated with vincristine once a week for 4 weeks (ALL-BFM 2009 protocol; **Figure 1A**). Severe hyponatremia occurred in 14.7% overall (**Table 2**), a rate higher than those in the cohorts from Poland (11.9%) (24) and India (10.8%) (25). The average age of hyponatremic patients was 9.0 years; the average age of non-hyponatremic patients was lower (5.9 years). Hyponatremic patients thus were significantly older than non-hyponatremic patients (p = 0.0369, **Table 2**). Divided according to age groups, three hyponatremic patients (21.4%) were aged <5 years, 7 (50%) 5–10 years, none 10–13 years, and 4 (28.6%) >13 years.

In addition, 10 of the 14 hyponatremic patients were male (71.4%), a proportion seemingly at substantial variance with that in our cohort overall (56 of 95; 58.9%). However, within the cohort as a whole no statistically significant difference could be shown regarding gender (p = 0.385, **Table 2**), which may reflect the general preponderance of males among ALL patients (28). Our study nonetheless suggested increased susceptibility to hyponatremia among males of primary school age. We did not identify the predisposition for hyponatremia among girls aged >10 years reported from India (25).

Published data on the incidence of hyponatremia in pediatric oncological practice are limited to children with ALL, even though vincristine is also used in other pediatric tumors. As several reports ascribe severe hyponatremia in oncological patients to vinca-alkaloid therapy (6, 12, 13, 16, 17, 20), we investigated whether this held for non-ALL patients, on whom reports are sparse, particularly in children (4), as with a case study of a 4-year-old boy with Wilms tumor and severe symptomatic hyponatremia and seizures after vincristine administration (29). Our study is the first to compare incidence of hyponatremia in patients with various pediatric tumors treated with alkaloids. We found that therapy-associated hyponatremia is unique to ALL, as episodes of hyponatremia during induction therapy did not occur in statistically significant numbers in patients with Ewing sarcoma (one vincristine bolus at start of therapy), with Hodgkin’s lymphoma, Wilms tumor, or benign CNS gliomas (weekly vincristine bolus during the first weeks of therapy), or Langerhans-cell histiocytosis (weekly vinblastine bolus during first weeks of therapy) (**Figure 2B** and **Table 3**). Vinca alkaloids thus do not appear *per se* to suffice as an etiology for hyponatremia.

What factors other than vinca alkaloids might contribute to hyponatremia in ALL that are absent or irrelevant in other malignancies? Janczar et al. reported an association between hyponatremia with neurologic symptoms following vincristine administration and CNS involvement with leukemia at diagnosis. They emphasized the role of cytostatic therapy rather than leukemia alone in severe hyponatremia, as no hyponatremic episode occurred before treatment with vincristine began (24). However, we found no association between leukemic CNS involvement and hyponatremia in ALL patients, as only 2 of our 14 hyponatremic patients had with blasts in cerebrospinal fluid at presentation (p = 0.274, **Table 2**). Nor could we state that high-risk ALL potentiates the development of hyponatremia during induction therapy, as only 5 of our 14 hyponatremic patients met high-risk criteria (35.7%, p = 0.292, **Table 2**).

We examined in our ALL cohort various biomarkers that might reflect susceptibility to hyponatremia during vincristine therapy. Serum sodium values may erroneously be assessed as low in patients with hypertriglyceridemia, hyperproteinemia, or hyperglycemia when sodium is measured using indirect potentiometry (30–33). Most ALL patients in induction therapy, however, show pronounced hypoproteinemia due to diminution in protein synthesis associated with asparaginase therapy (34). Of our hyponatremic patients, 85.7% (12/14) showed pronounced hypoproteinemia (<5 g/dl) coincident with hyponatremia; 70.4% (57/81) of our non-hyponatremic patients had serum protein levels <5 g/dl during induction therapy. No patient showed hyperproteinemia at that time (**Table 2**). Moreover, only 2/14 (14.3%) hyponatremic patients had hyperglycemia coincident with hyponatremia, whereas 28/81 (34.6%) non-hyponatremic patients were hyperglycemic during induction therapy (p = 0.767, **Table 2**). We thus infer that hyperproteinemia and hyperglycemia were not responsible for artifactual hyponatremia, *viz.*, pseudohyponatremia, in our ALL cohort. Interestingly, in 42.9% (6/14) of our hyponatremic patients marked hypertriglyceridemia (>1,000 mg/dl) coincided with hyponatremia (**Table 2**). In another, a lesser degree of hypertriglyceridemia (<500 mg/dl) coincided with hyponatremia. In 4 (28.6%) of our 14 hyponatremic patients, no serum triglyceride values during induction therapy were available. However, two of these four triglyceride values rose markedly during reinduction therapy. We suspect that the percentage of hyponatremic patients with hypertriglyceridemia may have been higher than detected, especially in view of evidence that the incidence of hypertriglyceridemia in ALL patients is relatively high (35). As triglyceride values >1 000 mg/dl were seen in only 4.9% (4/81) of non-hyponatremic patients, but in 42.9% (6/14) of hyponatremic patients (**Table 2**), we could demonstrate a statistically highly significant association between massive hypertriglyceridemia and the occurrence of hyponatremia in ALL patients during induction therapy (p = 0.00017, **Table 2**).

In our department, nearly all newly diagnosed patients, especially those with great tumor burden (ALL, but also other entities such as Hodgkin’s lymphoma, Wilms tumor, and Ewing sarcoma), receive intravenous semi-isotonic hydration (see *Materials and Methods*) as tumor lysis syndrome prophylaxis. Administration of hypotonic maintenance fluids may be associated with a greater risk for hyponatremia than is administration of isotonic solutions (36–40). Hyponatremia in our patients thus might conceivably have reflected serum dilution. Against this are, first, that hyponatremia was seen mainly in the ALL cohort and not in similarly treated patients with other diagnoses, and second, that infusion therapy is usually administered only for the first few days—up to at most 1 week—after induction chemotherapy begins. However, hyponatremic episodes were detected days later, as the decline in sodium levels was statistically significant only from day 12 after therapy began (**Figure 2A**, p = 0.014, **Supplemental Table**), precluding dilutional artifact. Not only this: the prevalence of hyponatremia is still high despite use of recommended isotonic fluid regimes (41).

Certain medications used simultaneously with vinca alkaloids may worsen the risk for hyponatremia in induction therapy for ALL (27). However, hyponatremia has not yet been ascribed to other cytostatic agents used during Protocol I of induction therapy of the ALL-BFM 2009 study (**Figure 1A**). Known possible potentiators are azole antifungals (itraconazole, voriconazole, posaconazole, ketoconazole) (42–47). These inhibit catabolism of vinca alkaloids, increasing exposure. Only high-risk patients in our ALL cohort received antifungal prophylaxis, however, and with liposomal amphotericin-B rather than azoles. Azole potentiation of hyponatremia thus cannot be invoked as contributory to hyponatremia in our patients.

Nor can the type of therapy regimen with vinca alkaloids be the only cause of the high incidence of hyponatremia in childhood ALL. The weekly administration of 1.5 mg/m^2^/day (maximum 2 mg) vincristine as an intravenous bolus is absolutely comparable in dose and frequency to that used in therapy regimens for other malignancies, regimens that in this study were not associated with episodes of hyponatremia. The first hyponatremic episode in our ALL cohort started on average between days 27 and 28 (average day 27.6) but occurred earlier or later in some patients (range from day 12 to day 39 after start of therapy, **Figure 2A**). This is consonant with others’ observations, which place the onset of hyponatremia approximately 7 days or even longer after receiving vincristine intravenously (7, 9, 16, 48, 49). We propose, as suggested elsewhere (17), that development of marked hyponatremia associated with vinca alkaloids definitely requires repeated administration of vincristine. A single dose may not be enough to cause severe hyponatremia (for example, in patients with Ewing sarcoma).

In conclusion, we here show a substantial incidence of hyponatremia in a pediatric ALL cohort during induction chemotherapy with weekly administered vincristine. Hyponatremic episodes were more likely in males of primary school age. Occurrence of hyponatremia was not statistically significant in cohorts of children with other malignancies and comparable vinca-alkaloid therapy. None of our patients showed hyponatremia-associated severe neurological symptoms or complications, perhaps because sodium levels fell only moderately (least observed, 123 mmol/l). In addition, some instances of hyponatremia were certainly factitious (pseudohyponatremia), in part an artifact of hypertriglyceridemia. This might explain our patients’ lack of neurological complications. We accordingly recommend that serum triglycerides be determined regularly during ALL induction therapy to permit assessment of whether hyponatremia is artifactual (and can be disregarded) or true (and warrants intervention).

## Data Availability Statement

The data analyzed in this study are subject to the following licenses/restrictions: retrospective evaluation. Requests to access these datasets should be directed to the PowerChart Program of State Hospital of Innsbruck.

## Ethics Statement

The studies involving human participants were reviewed and approved by the Ethics Committee of the Medical University of Innsbruck. Written informed consent from the participants’ legal guardian/next of kin was not required to participate in this study in accordance with the national legislation and the institutional requirements.

## Author Contributions

CS contributed to the conception and design of the retrospective study, reviewed the literature for data, and wrote the manuscript. RS was responsible for the statistical analysis and critical interpretation of data. CK and AH contributed to the acquisition of patients’ data. GK, TM, PW and RC critically revised the manuscript for important intellectual content. All authors contributed to the article and approved the submitted version.

## Conflict of Interest

The authors declare that the research was conducted in the absence of any commercial or financial relationships that could be construed as a potential conflict of interest.

## Publisher’s Note

All claims expressed in this article are solely those of the authors and do not necessarily represent those of their affiliated organizations, or those of the publisher, the editors and the reviewers. Any product that may be evaluated in this article, or claim that may be made by its manufacturer, is not guaranteed or endorsed by the publisher.
